# Molecular characterization and gene expression modulation of the alternative oxidase in a scuticociliate parasite by hypoxia and mitochondrial respiration inhibitors

**DOI:** 10.1038/s41598-020-68791-9

**Published:** 2020-07-17

**Authors:** Iría Folgueira, Jesús Lamas, Rosa Ana Sueiro, José Manuel Leiro

**Affiliations:** 1 Department of Microbiology and Parasitology, Laboratory of Parasitology, Research Institute on Chemical and Biological Analysis, University of Santiago de Compostela, Campus Vida, 15782 Santiago de Compostela, Spain; 2 Department of Fundamental Biology, Institute of Aquaculture, University of Santiago de Compostela, Campus Vida, 15782 Santiago de Compostela, Spain

**Keywords:** Microbiology, Parasitology, Parasite genomics

## Abstract

*Philasterides dicentrarchi* is a marine benthic microaerophilic scuticociliate and an opportunistic endoparasite that can infect and cause high mortalities in cultured turbot (*Scophthalmus maximus*). In addition to a cytochrome pathway (CP), the ciliate can use a cyanide-insensitive respiratory pathway, which indicates the existence of an alternative oxidase (AOX) in the mitochondrion. Although AOX activity has been described in *P. dicentrarchi*, based on functional assay results, genetic evidence of the presence of AOX in the ciliate has not previously been reported. In this study, we conducted genomic and transcriptomic analysis of the ciliate and identified the AOX gene and its corresponding mRNA. The AOX gene (size 1,106 bp) contains four exons and three introns that generate an open reading frame of 915 bp and a protein with a predicted molecular weight of 35.6 kDa. The amino acid (aa) sequence of the AOX includes an import signal peptide targeting the mitochondria and the protein is associated with the inner membrane of the mitochondria. Bioinformatic analysis predicted that the peptide is a homodimeric glycoprotein, although monomeric forms may also appear under native conditions, with EXXH motifs associated with the diiron active centers. The aa sequences of the AOX of different *P. dicentrarchi* isolates are highly conserved and phylogenetically closely related to AOXs of other ciliate species, especially scuticociliates. AOX expression increased significantly during infection in the host and after the addition of CP inhibitors. This confirms the important physiological roles of AOX in respiration under conditions of low levels of O_2_ and in protecting against oxidative stress generated during infection in the host.

## Introduction

Alternative oxidase (AOX), also known as ubiquinol-oxidoreductase, is included within the diiron carboxylate superfamily. The enzyme is characterized by cyanide resistance and is associated with maintenance of metabolic homeostasis via mitochondrial electron transport in plants subjected to various stress conditions^1,2^. In plants, AOX is considered a ubiquitous enzyme; however, in algae^3^, fungi^4–9^, protozoa^10–13^ and several invertebrate species^14^ it is quite sporadic. AOX is involved in the mitochondrial respiratory chain and provides an alternative pathway for the direct transfer of electrons from ubiquinone to oxygen, thus forming water^15^. It facilitates mitochondrial respiratory activity and therefore the functioning of the Krebs cycle after inhibition of the cytochrome route due to the presence of nitric oxide, cyanide (both inhibitors of the complex IV) or antimycin A (complex III inhibitor)^1,12,16^. However, AOX reduces the energy efficiency of respiration as only 1/3 of the ATP is produced by pumping protons through complex I^17^. In plants, AOX participates in important biological processes, such as survival and adaptation to stress factors in biotic and abiotic environments, resistance to oxidative stress and maintenance of mitochondrial and cellular energy homeostasis^18,19^. In parasitic protists, it is particularly associated with metabolic adaptation to the host and pathogenicity^20–22^. AOX is present in the mitochondria of protists such as the amoebozoans *Acanthamoeba castellani*^23^, *A. polyphaga*^24^ and *Dictyostelium discoideum*^25^, *Trypanosoma brucei*^26^*, T. congolensi* and *T. evansi*^27^, *Plasmodium falciparum*^28^, *Cryptosporidium parvum*^29^ and *Blastocystis hominis*^16,22^. AOX has also been detected in the Euglenozoa *Euglena gracilis*^30^, in the ciliates *Paramecium tetraurelia*^31^, *Tetrahymena pyriformis*^32^ and *Ichthyophthirius multifiliis*^33^; and in the scuticociliate parasites *Pseudocohnilembus persalinus*^34^ and *Philasterides dicentrachi*^13^. In previous studies, we have demonstrated the presence of a cyanide-resistant alternative respiratory pathway (AP) in mitochondria of the scuticociliate parasite *P. dicentrarchi*, in addition to the conventional cytochrome pathway (CP)^13^. This provided physiological evidence for the presence of a cyanide-insensitive oxidase, which was recognised by antibodies raised against a synthetic peptide derived from a fully conserved C-terminal consensus motif from plant AOX isoforms^13^. In addition, AOX inhibitors such as salicylhydroxamic acid (SHAM) and 3,4,5-trihydroxybenzoic acid (propyl gallate, PG) significantly inhibit in vitro growth of the ciliate^13,35,36^.

Although there is clear physiological evidence for the existence of an AOX in *P. dicentrarchi*, this enzyme has not previously been identified or characterized in detail at the molecular level. In the present study, we characterized the *P. dicentrarchi* AOX gene and detected the AOX protein in the mitochondria. We also investigated the transcriptional modulation of this enzyme in vitro, in response to experimentally generated hypoxia, as well as in response to the presence of inhibitors of CP and AP mitochondrial respiration.

## Materials and methods

### Experimental animals and ethics statement

Turbot (*Scophthalmus maximus*). Juvenile fish (50 g) were obtained from a fish farm in Galicia (NW Spain). The fish were held in 200 L fibreglass tanks with a water recirculation system, at 16 °C with constant aeration and a photoperiod of 12L:12D, and were fed daily with commercial pellets (Skretting, Burgos, Spain). The fish were acclimatized to the aquarium conditions for 2 weeks before the start of the experiments.

Mice. Female CD-1 mice (Charles River Laboratories, USA) of weight 25 g were supplied by the Central Animal Husbandry Unit of the University of Santiago de Compostela (Spain).

All animal experiments and protocols were conducted in accordance with Spanish and European Legislation (R.D. 53/2013 and Council Directive 2010/63/EU) and approved by the Institutional Animal Care and Use Committee of the University of Santiago de Compostela (Spain). When required during all experiments, the animals were anaesthetized with isoflurane (mice) or 100 mg/L of tricaine methane sulphonate-MSS-222-(turbot) and finally euthanized by decapitation (mice) or by overdose of anaesthesia (turbot).

### Cell culture and experimental infections

The isolate of *P. dicentrarchi*^37^ used (I1) was obtained from ascites of experimentally infected turbot (*Scophthalmus maximus*)^38^. The isolate was grown axenically at 21 °C in complete L-15 medium^39^ under (a) normoxic conditions, in culture flasks provided with vented caps to allow aeration of the culture medium, or (b) under hypoxic conditions, by bubbling the culture flasks for 10 min with argon gas and tightly closing the flask with non-vented caps, after addition or removal of the ciliates^13^.

### Sequencing of the genome and transcriptome

For analysis of the *P. dicentrarchi* genome and transcriptome, trophonts (10^7^) were concentrated by centrifugation, frozen in liquid nitrogen and sent on dry ice to Future Genomic Technologies (Leiden, Netherlands). For sequencing of the complete genome of the ciliate, a combination of short reading sequencing (Illumina technology) and long reading sequencing (Nanopore technology) was used (Oxford Nanopore Technologies). For de novo assembly of the parasite genome, the data sets were combined using the TULIP program v0.4^40^. Transcript sequences from Illumina RNA- Seq data (fragments of approximately 100 bp), obtained by amplification by SBS, were assembled using Trinity software (v2.6.5)^41^, included within the Galaxy application (https://usegalaxy.org/). The assembled sequences were analyzed by homology, with Blastgo 5.0 software (Biobam, Spain), and annotated. The sequences that encode proteins that are potentially related to the ciliate AOX were then selected from the *Tetrahymena thermophila* gene and protein sequences database by using the BLASTx tool Wiki TGD (https://www.ciliate.org/blast/blast_link.cgi).

### Production of recombinant AOX (rAOX) in yeast cells

The complete nucleotide sequence that encodes *P. dicentrarchi* AOX was obtained with an open reading frame (ORF) search tool (ORF Finder; https://www.ncbi.nlm.nih.gov/orffinder/), from the annotated data obtained by analysis of a RNA-Seq experiment. The codons of the original nucleotide sequence in *P. dicentrarchi* were optimized using the Integrate DNA Technologies (IDT) bioinformatics tool (https://eu.idtdna.com/CodonOpt), to produce a recombinant protein in the yeast *Klyuveromyces lactis*. The AOX gene nucleotide sequence was synthesized by Invitro GeneArt Gene Synthesis (ThermoFisher Scientific), amplified by PCR with the primers FaoxKl / RaoxKl: 5′-CGC CTC GAG AAA AGA ATG CAG TCC TTC GCC AGG AAA-3′/5′-ATA AGA ATG CGG CCG C TTA GTG GTG GTG ATG ATG GTG ATG ATG ATG ATG CTG CTC GGG GGA GTA AGG GTT 3′ and cloned in the integrative expression vector pKLAC2. The *K. lactis* Protein Expression kit (New England Biolabs, UK) was used to express the recombinant protein in yeast according to the manufacturer’s instructions and the protein secretion strategy, as previously described^42^.

### Obtaining anti-rAOX immune serum

The anti-rAOX antiserum used in the immunoassays was obtained from CD-1 mice immunized intraperitoneally (i.p.) with 200 µg of rAOX. The antiserum was purified by immobilized metal affinity chromatography (IMAC) with Ni-Sepharose^42^ and adsorbed for 30 min at room temperature in 200 µL of a 1% chitosan hydrogel adjuvant^42^ (CH) (w/v) in PBS buffer, pH 7.0. At intervals of 15 and 30 days after the first immunization, mice were reimmunized with the same dose of rAOX and adjuvant. Seven days after the second immunization, the mice were bled by decapitation and the blood thus obtained could clot overnight at 4° C. The serum was clarified by centrifugation at 2000 × *g* for 10 min, mixed 1: 1 (v/v) with glycerol, and stored at −20 °C until use.

### Sodium dodecyl sulphate polyacrylamide gel electrophoresis (SDS-PAGE) and Western-blotting

SDS-PAGE of the rAOX protein and a ciliate lysate (CL)—obtained from cultures maintained under normoxic or hypoxic conditions^44^—and treated under reducing conditions (after the addition of 0.02 M dithiothreitol-DTT-) or under non-reducing (without the addition of DTT), was performed on linear 12.5% polyacrylamide minigels in a Mini-Protean Tetra cell system (BioRad, USA), as previously described^35^. Once electrophoresis was completed, the gels were stained with a solution of GelCode Blue Stain Reagent (Thermo Scientific) according to the manufacturer's instructions.

Samples separated by electrophoresis were also analyzed by Western blot, with a slightly modified version of a previously described protocol^35^. The samples were first incubated with serum from mice immunized with AOX (anti-rAOX serum) and then with a polyclonal peroxidase-conjugated rabbit anti-mouse antibody (Dakopatts, Denmark) at 1:800 dilution. The blots were stained by adding a chromogenic enzyme substrate solution consisting of 0.003% H_2_O_2_ and 0.06% 3,30-diaminobenzidine tetrahydrochloride with 0.03% NiCl2 (DAB/NiCl2, Sigma, USA).

### Indirect immunofluorescence (IIF)

For AOX immunolocalization in trophonts, an IIF immunoassay was performed, as previously described^46^. Briefly, ciliates were fixed in a solution of 4% formaldehyde in PBS, permeabilized in a solution containing 0.3% Triton X-100 in PBS and blocked with a solution of 1% BSA. The ciliates were then incubated with a mouse antiserum containing anti-rAOX antibodies diluted 1:100 in PBS and with a secondary polyclonal rabbit anti-mouse immunoglobulin conjugated with fluorescein isothiocyanate (FITC; DAKO, Denmark) and diluted 1:1,000. The ciliates were then visualized by fluorescence microscopy (Zeiss Axioplan, Germany).

### Transmission electron microscopy (TEM)

For TEM analysis we followed the technique described by Paramá et al.^47^. Briefly, the cultured ciliates were fixed in 2.5% (v/v) glutaraldehyde in 0.1 M cacodylate buffer at pH 7.2, post-fixed in 1% (w/v) OsO4, pre-stained in saturated aqueous uranyl acetate, dehydrated in acetone series and embedded in Spurr’s resin. Ultrathin sections were stained with 2.5% uranyl acetate in 50% ethanol and 0.5% of lead citrate in 4.5 mM NaOH and viewed in a Jeol JEM-1011 transmission electron microscope (Jeol, Japan) at an accelerating voltage of 100 kV.

### In vitro growth assay

To determine the growth of the ciliates under conditions of normoxia and hypoxia, we followed the protocol described by Mallo et al.^13,35^. Briefly, the ciliates were cultured at an initial concentration of 5 × 10^4^ trophonts/mL in complete L-15 medium in culture flasks fitted with vented caps that allowed aeration of the culture medium (normoxic conditions) and in tightly closed bottles bubbled with argon (hypoxic conditions). To ensure hypoxic conditions, the L-15 medium was bubbled with argon for 10 min until gas saturation, to completely displace the dissolved oxygen in the medium. The ciliates were then added, and the bottles were hermetically sealed to prevent entry of oxygen. To determine growth of the cultures under conditions of normoxia and hypoxia, aliquots of 100 μL were collected daily for 6 days and the number of ciliates was quantified in a Neubauer counting chamber^48^. After collection of each sample to quantify the ciliates, the argon bubbling was repeated in all cases under the same conditions as described above to maintain the hypoxic conditions.

### Reverse transcriptase-quantitative polymerase chain reaction (RT-qPCR)

The RT-qPCR technique was basically performed as previously described, with slight variations^35^. Briefly, the total RNA extracted from 10^6^ trophozoites/mL of *P. dicentrarchi* cultured under hypoxic/normoxic conditions, in the presence of 1 mM SHAM and 1 mM KCN (inhibitors of AOX and cytochrome-complex IV-, respectively), was isolated with a NucleoSpin RNA isolation kit (Macherey–Nagel) according to the manufacturer’s instructions. The quality, purity and concentration of RNA were quantified in a NanoDrop ND-1000 Spectrophotometer (NanoDrop Technologies, USA). Two μg of sample RNA, 1·25 μM random hexamer primers (Promega) and 200 U of Moloney murine leukemia virus reverse transcriptase (MMLV; Promega) were used for cDNA synthesis (RT). Quantitative polymerase chain reaction (qPCR) analysis was performed with AOX gene-specific primers forward/reverse pair FqAOX/RqAOX (5′ TCG GAG ACT CCT TCG CTT AC-3′/5′-CCA TCC TTG GTC TCT TTC CA-3′) and *P. dicentrarchi* elongation factor 1-alpha gene (EF-1α) (GenBank accession KF952262) forward/reverse primer pair (FEF1A/REF1A: 5′-TCG CTC CTT CTT GCA TCG TT-3′/5′-TCT GGC TGG GTC GTT TTT GT-3′) as housekeeping gene. The reaction mixture (10 μL) used in the qPCR contained 5 μL of PowerUp SYBR Green Master Mix (Applied Biosystems), 300 nM of the primer pair, 1 μL of cDNA and RNase–DNase-free water. The qPCR was run at 95 °C for 5 min, followed by 40 cycles at 95 °C for 10 s and 60 °C for 30 s, ending with melting-curve analysis at 95 °C for 15 s, 55 °C for 15 s and 95 °C for 15 s. in a StepOnePlus Real Time System (Thermo Fisher Scientific). Finally, relative quantification of gene expression was determined by the 2^−ΔΔct^ method^49^ with the software included in the equipment.

### Bioinformatic and statistical analysis

Functional analysis of proteins and classification into different families to predict the domains and important sites was carried out with INTERPRO software^50^. The transmembrane topology and location of signal peptide cleavage sites in amino acid (aa) sequences were predicted with the PHOBIUS program^51^. The N-terminal region of the protein that may contain a mitochondrial signal sequence and its cleavage site were analyzed with the MITOPROT II -v1.101 program^52^. The physicochemical parameters were predicted for a given protein with the The PROTPARAM tool^53^. The O-ß-GlcNAc attachment sites were predicted with the YINGOYANG^54^ and OGLCNACSCAN^55^ programs. For visualization of proteoforms and integration of the protein topology, the PROTTER bioinformatics tool was used^56^. Sub-cellular localization was predicted with the LOCTREE3 program^57^. The SWISS-MODEL protein server was used for modelling protein structures and complexes^58^. The aa sequences were aligned using the CLUSTAL OMEGA multiple sequence alignment program ^59^, and the phylogenetic signal/noise ratio was improved with the TRIMAI tool for automated alignment trimming^60^. The phylogenetic trees were constructed by the Maximum Likelihood (ML) method, with the JTT model^61^. Branch support was given with 1,000 bootstrap replicates^62^ in MEGAX software^63^. The Bayesian inference (BI) analysis was performed with MRBAYES 3.2.6^64^.

The values shown in the text and figures are means ± SEM. One-way analysis of variance (ANOVA) was used for comparison of more than two samples, and the Tukey-Kramer test was used for pairwise comparisons. The Student’s t-test was used for comparison of two samples. In all cases, differences were considered significant at *P* < 0.05.

## Results

### Overall structure of AOX in *P. dicentrarchi*

**Figure 1:**
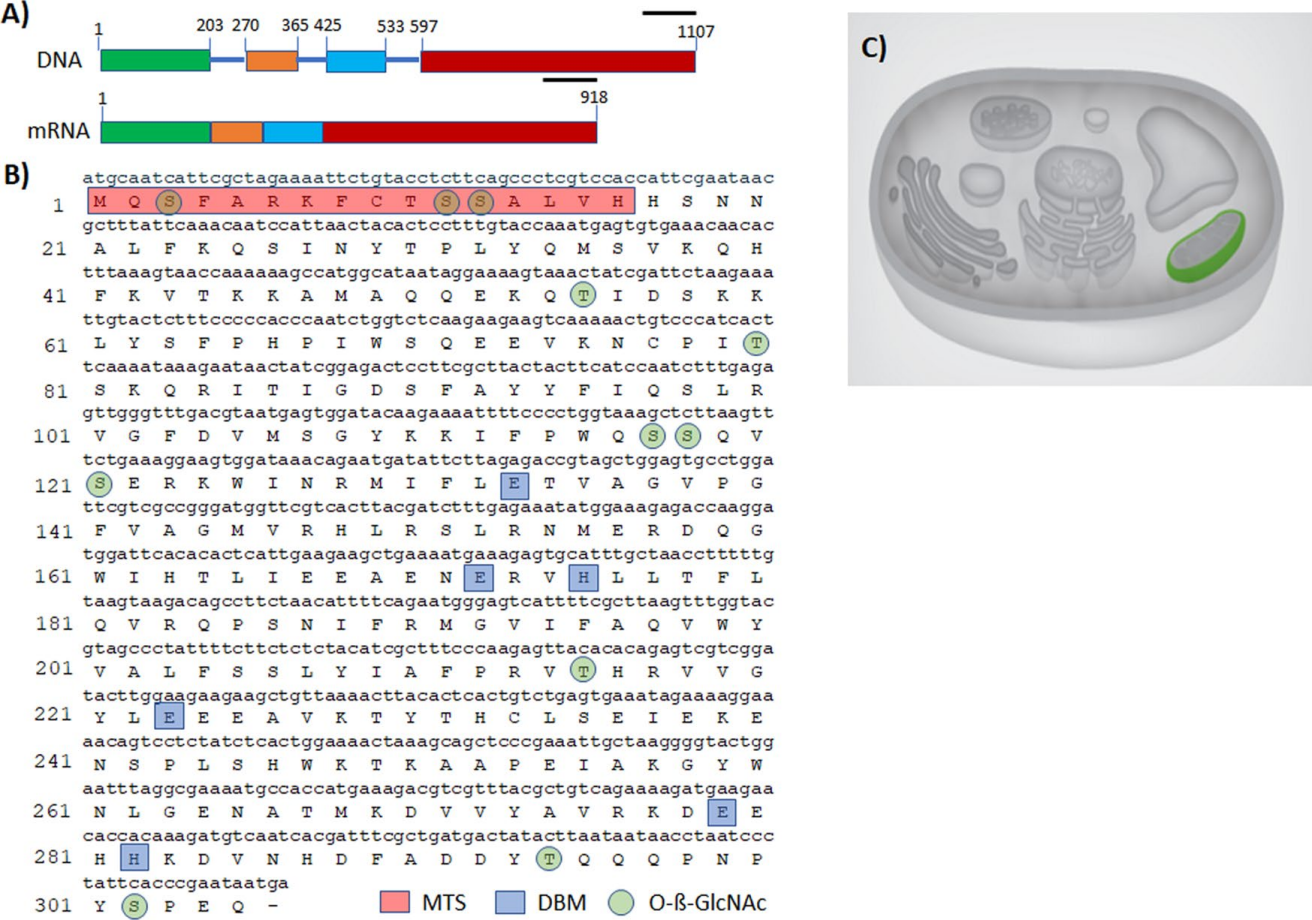
(**A**) Schematic representation of the structure of the AOX gene of *P. dicentrarchi*, indicating the position of exons (squares) and introns (lines) in the nucleotide sequence, as well as their translation to mRNA. Linear scale = 100 nucleotides (**B**) *Philasterides dicentrarchi* AOX mRNA, complete cds (GenBank: MH427340.1), together with its corresponding translation into amino acids (GenBank: QAR17767.1), including the mitochondrial targeting sequences (MTS), the diiron binding motifs [ion binding site] (DBM), and predictions for O-ß-GlcNAc attachment sites. (**C**) Diagram of the bioinformatic prediction of the cellular location of the amino acid sequences of the AOX provided by the LocTree 3 program (Rostlab, Technical University of Munich, Germany; URL: https://rostlab.org/services/loctree3/).

The complete sequences of the AOX gene and cDNA were deposited in the NCBI GenBank database, with accession numbers MN193569 and MH427340, respectively. The gene encoding the AOX has a total length of 1,107 bp and contains 4 exons and 3 introns, with an average intron size of around 60 bp (Fig. 1A). The coding sequence contains 918 bp that encodes a protein of 305 aa with an estimated molecular mass of 35,638.68 daltons and a theoretical pI of 9.03. Bioinformatic analysis also indicated that this protein lacks a signal peptide; however, the MitoPro II program predicted the existence of an import signal peptide targeting the mitochondria MQSFARKFCTSSALV with a probability of 0.9503 and a cleavage site in aa 16 (Fig. 1B), as also confirmed by the bioinformatic prediction of cellular localization in the mitochondria through the LocTree program (Fig. 1C). Bioinformatic analysis with the InterPro program (which provides functional analysis of proteins by classifying them in families and predicts important domains and sites in the sequence) showed that the protein belongs to the AOX family, i.e. that it has 6 diiron binding motifs and is O-glycosylated in the aa S3, T10, S11, T55, S117, S118, S121, T215, T259, S302 (Fig. 1B). Prediction of the biological function from the amino acid sequence of the AOX to establish the gene ontology (GO) terms indicates that this protein is associated with oxidation–reduction processes and with a biological function involving alternative oxidase activity. Analysis of the topology of the aa sequence using the Phobius bioinformatic program revealed that this protein has two transmembrane regions associated with the mitochondrial inner membrane between aa 125 and 146 and 188–211, a region located between aa 1–125 and between aa 212–305 that is located inside the mitochondrial matrix, and a region located between aa 147 and 187 that is located towards the intermembrane space of the mitochondria. However, the program for predicting transmembrane helices in proteins (TMHMM-ver. 2.0) only identified a single transmembrane region associated with the mitochondrial inner membrane between aa 188–211, a region between aa 1–188 that is located inside the mitochondrial matrix, and a region located between aa 212–305 directed towards the mitochondrial intermembrane space.

### Structure of diiron active site of AOX

**Figure 2:**
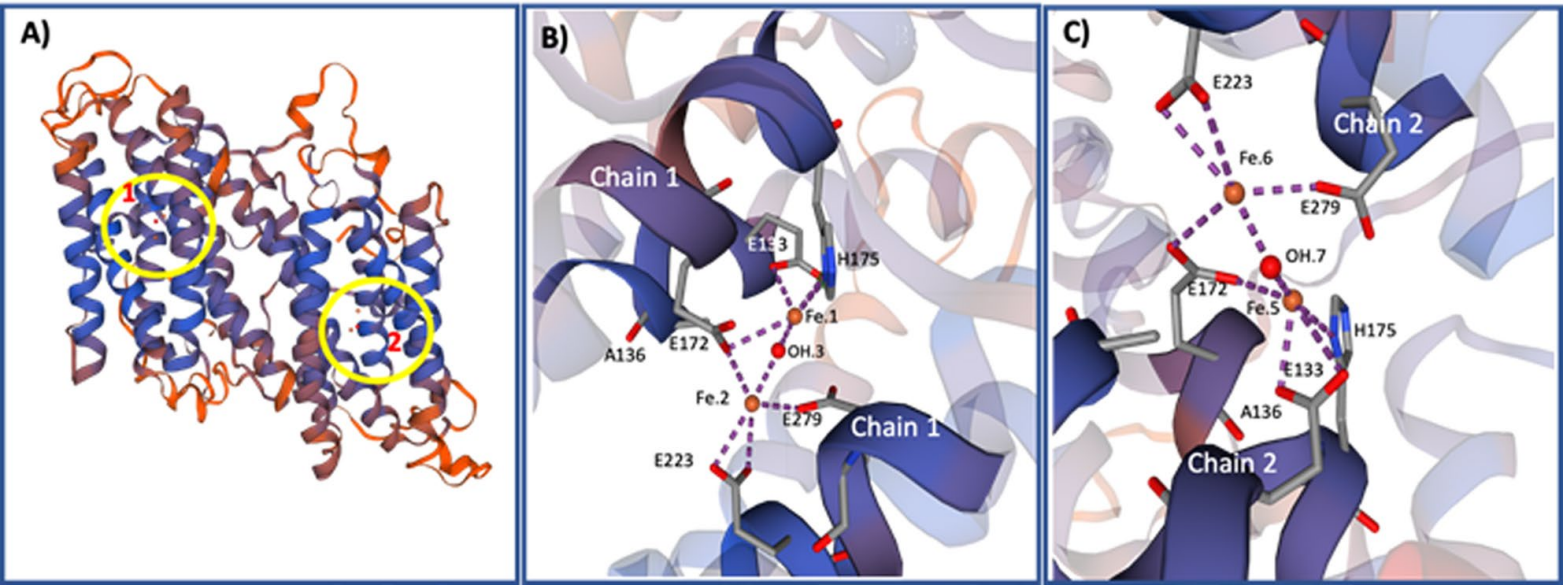
Structural modelling and active sites of *P. dicentrarchi* AOX. (**A**) Model of spatial structure for AOX homodimeric complex showing regions where the active sites containing the diiron binding motifs in the two polypeptide chains (1 and 2) of the enzyme are located (circles). (**B**) Diiron active site in chain 1, showing diiron and hydroxo atoms as well as the interactions of the diiron complex with the glutamate and histidine residues. (**C**) Diiron active site in chain 2, showing diiron and hydroxyl atoms as well as the interactions of the diiron complex with the glutamate and histidine residues. In both cases, the diiron site (iron moieties in orange, hydroxyl in red) is contained within a four alpha-helix bundle. The images of build model were generated by the Swiss-Model program (BIOZENTRUM, The Centre for Molecular Life Sci, University of Basel, Switzerland; URL: https://swissmodel.expasy.org/).

The protein structure modelling of AOX generated by the Swiss-Model and Swiss-PBD Viewer programs showed that the protein is homodimeric (Fig. 2A) and that each monomer contains an active site formed by two diiron centers and a OH ion (Fe^3+^–OH–Fe^3+^) (Fig. 2A,B). The computational ligand modeling indicated that Fe.1 has three ligand contacts with aa, two with glutamate (E133, E172) and one with histidine H175) residues, and one with OH; while Fe.2 interacts with three glutamate residues (E172, E223 and E279) and with OH group. Similarly, the active site of the other monomer also contains two diiron centers that interact with an OH group and with the same glutamate and histidine residues located in that monomer (Fig. 2C).

### Phylogenetic analysis

**Figure 3:**
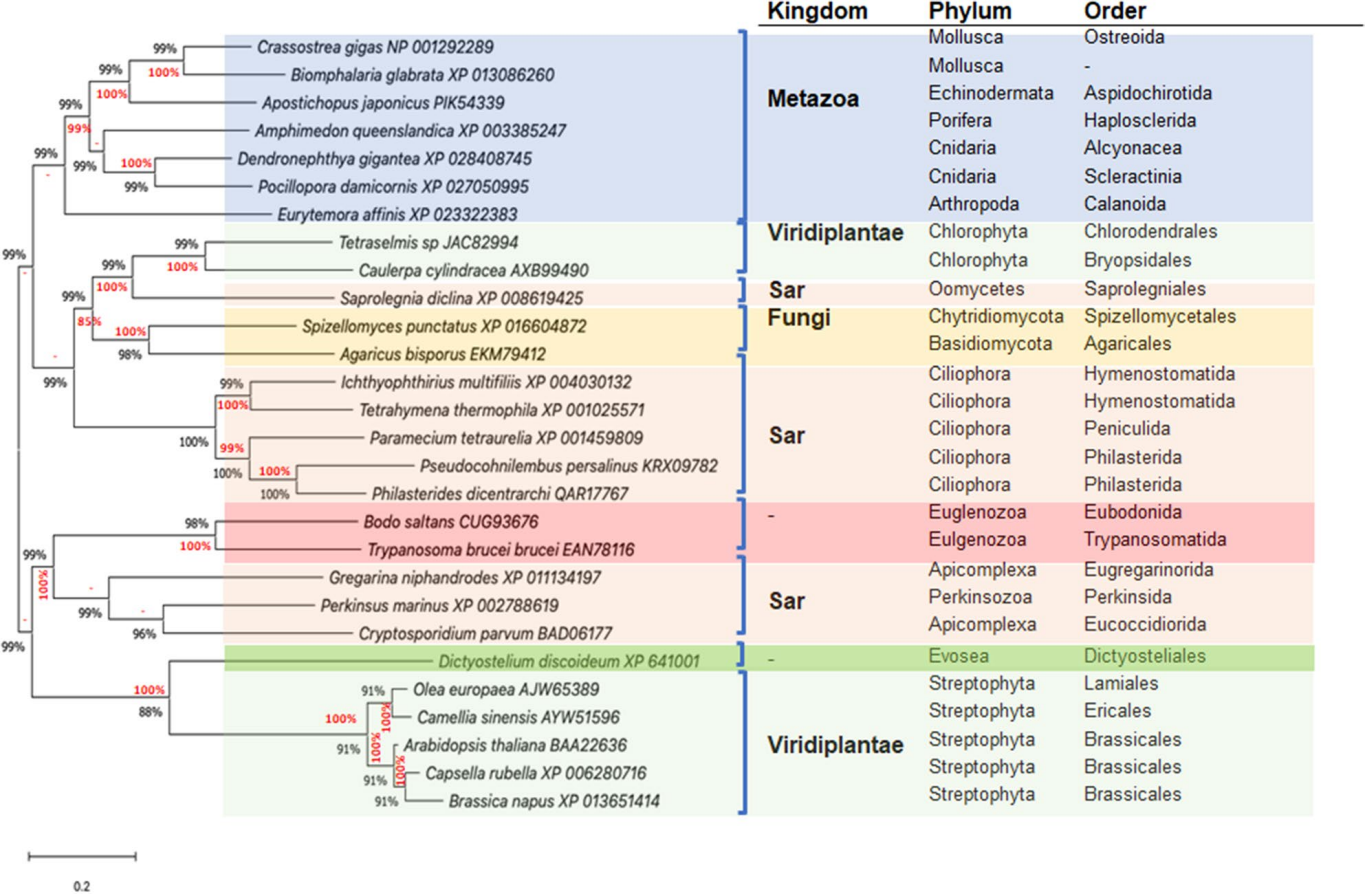
Phylogenetic analysis of amino acid sequences of *P. dicentrarchi* AOX inferred by Maximum Likelihood method (ML) and Bayesian inference (BI) methods, respectively. The analysis includes sequences of AOXs from representative species of Metazoa, Viridiplantae, Fungi and SAR. The GenBank access number is included after the name of the species. The tree with the highest log likelihood (− 8,244.09) is shown. The percentage of replicate trees in which the associated taxa clustered together in the bootstrap test (1,000 replicates) are shown beside the branches and represent the bootstrap value for ML analysis (black) and posterior probability value of BI analysis (red). Dashes (–) indicate disagreement between the ML and BI analysis. The tree is drawn to scale, with branch lengths in the same units as those of the evolutionary distances used to infer the phylogenetic tree. The evolutionary distances were computed using the Poisson correction method and are in the units of the number of amino acid substitutions per site. Analysis involved 28 amino acid sequences. All positions containing gaps and missing data were eliminated using the trimAl program. In total, 226 positions were included in the final dataset. Evolutionary analyses were conducted with the MEGAX and MrBayes programs. The scale bar corresponds to 20 substitutions per 100 aa positions.

We initially aligned the AOX amino acid sequences of three representative *P. dicentrarchi* strains (I1, B1 and C1) in order to estimate the degree of identity (Supplementary information). The AOX sequences in these *P. dicentrarchi* strains are highly conserved: there is a 100% identity between the AOX sequences of strains I1 and C1, and 99.67% identity with strain B1, which corresponds to a single change in a phenylalanine for a valine in aa 8 (Supplementary information). Analysis of the phylogenetic relationships between the aa sequence of the *P. dicentrarchi* AOX and those of AOXs of other Ciliophora indicate that are closely related to the AOX of the scuticociliate species *Pseudocohnilembus persalinus* (Fig. 3). The AOXs of Ciliophora species were also found to be more closely related to the AOX of Metazoan and Fungi than to Viridiplantae and other SAR species such as, for example, the Apicomplexa (Fig. 3).

### Biochemical analysis and subcellular localization of *P. dicentrarchi* AOX

**Figure 4:**
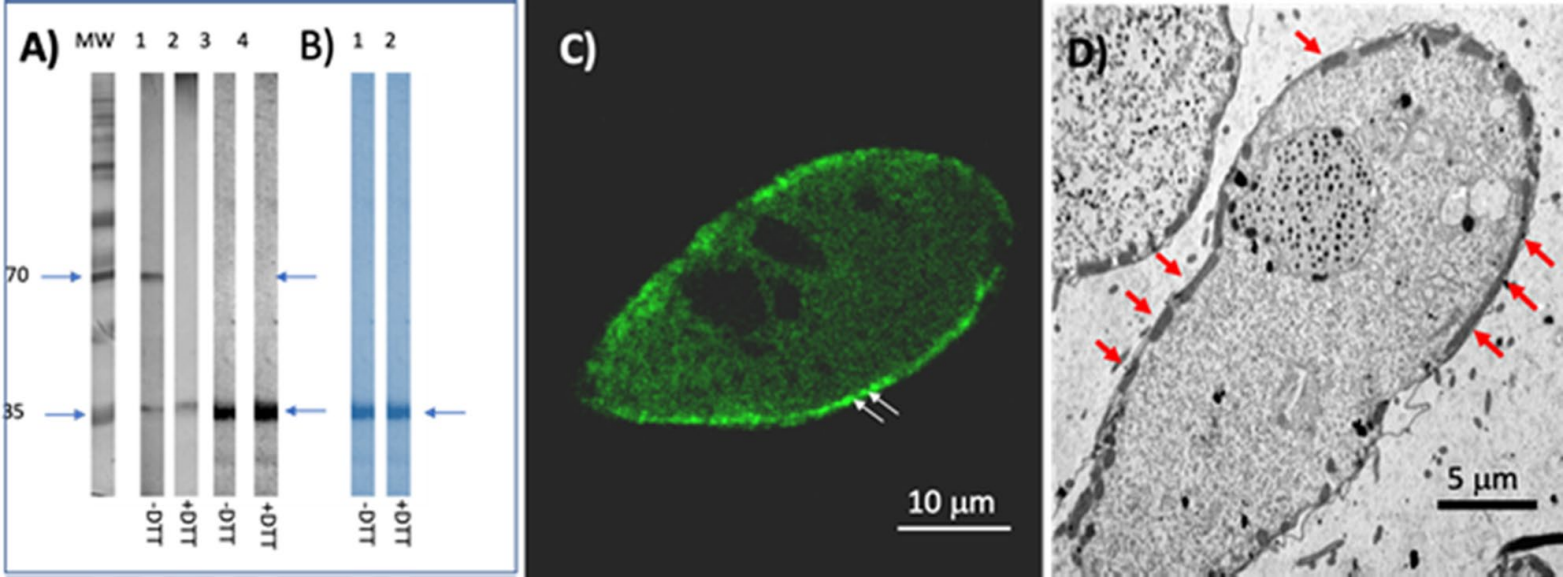
Biochemical characterization and cellular localization of the AOX of *P. dicentrarchi.* (**A**) Western blot analysis with a polyclonal anti-recombinant AOX (anti-rAOX) on a total ciliate lysate (CL, lanes 1 and 2) from trophonts of *P. dicentrarchi* maintained for 24 h in hypoxia and on the rAOX (lines 3 and 4). The proteins were separated by SDS-PAGE under non-reducing (− DTT, lanes 1 and 3) and reducing (+ DTT, lanes 2 and 4) conditions. (**B**) SDS-PAGE profile of rAOX stained with Coomassie blue under non-reducing (lane 1) and reducing conditions (lane 2). Arrows indicate the presence of recognition bands. *MW* molecular weight markers. (**C**) Representative photomicrograph of an immunofluorescence assay performed on a trophont of *P. dicentrarchi *and using a polyclonal anti-rAOX antibody in which intense fluorescent mitochondrial labelling is observed (arrows). (**D**) TEM microphotograph of a trophont of *P. dicentrarchi *showing the location of the mitochondria below the plasma membrane (arrows).

The biochemical properties of the AOX protein of *P. dicentrarchi* were investigated by SDS-PAGE and immunological techniques. To perform the biochemical analysis, a recombinant AOX was generated and expressed in the yeast *K. lactis*. The complete cDNA encoding the AOX was chemically synthesized and cloned in the yeast to produce the recombinant rAOX protein, which was purified and analyzed by SDS-PAGE, verifying the size at about 35 kDa (Fig. 4B, lanes 1 and 2). Once it was confirmed that the protein is expressed in yeast, a mouse polyclonal antibody was generated for use in immunological analysis. Western blot analysis with the anti-rAOX antibody verified the existence of a single recognition band on the recombinant protein (Fig. 4A) under both reducing (lane 4) and non-reducing conditions (lane 3). However, addition to the antibody of a complete lysate of the ciliate separated under reducing conditions (Fig. 4A, lane 2) or non-reducing conditions (Fig. 4A, lane 1) led to the presence of a single 35 kDa band, in the first case, and of two bands of 35 kDa (poorly stained) and 70 kDa (intensely stained), in the second case. This indicates that the enzyme, may appear in a monomeric or dimeric state (in its native state), in the latter case, of size 75 kDa. To demonstrate the cellular location of the AOX, we performed an indirect immunofluorescence assay using the polyclonal anti-rAOX antibody (Fig. 4C). This antibody generates intense labeling immediately below the plasma membrane at the location of the mitochondrion (Fig. 4D).

### Effect of hypoxia and mitochondrial respiration inhibitors on AOX expression

**Figure 5:**
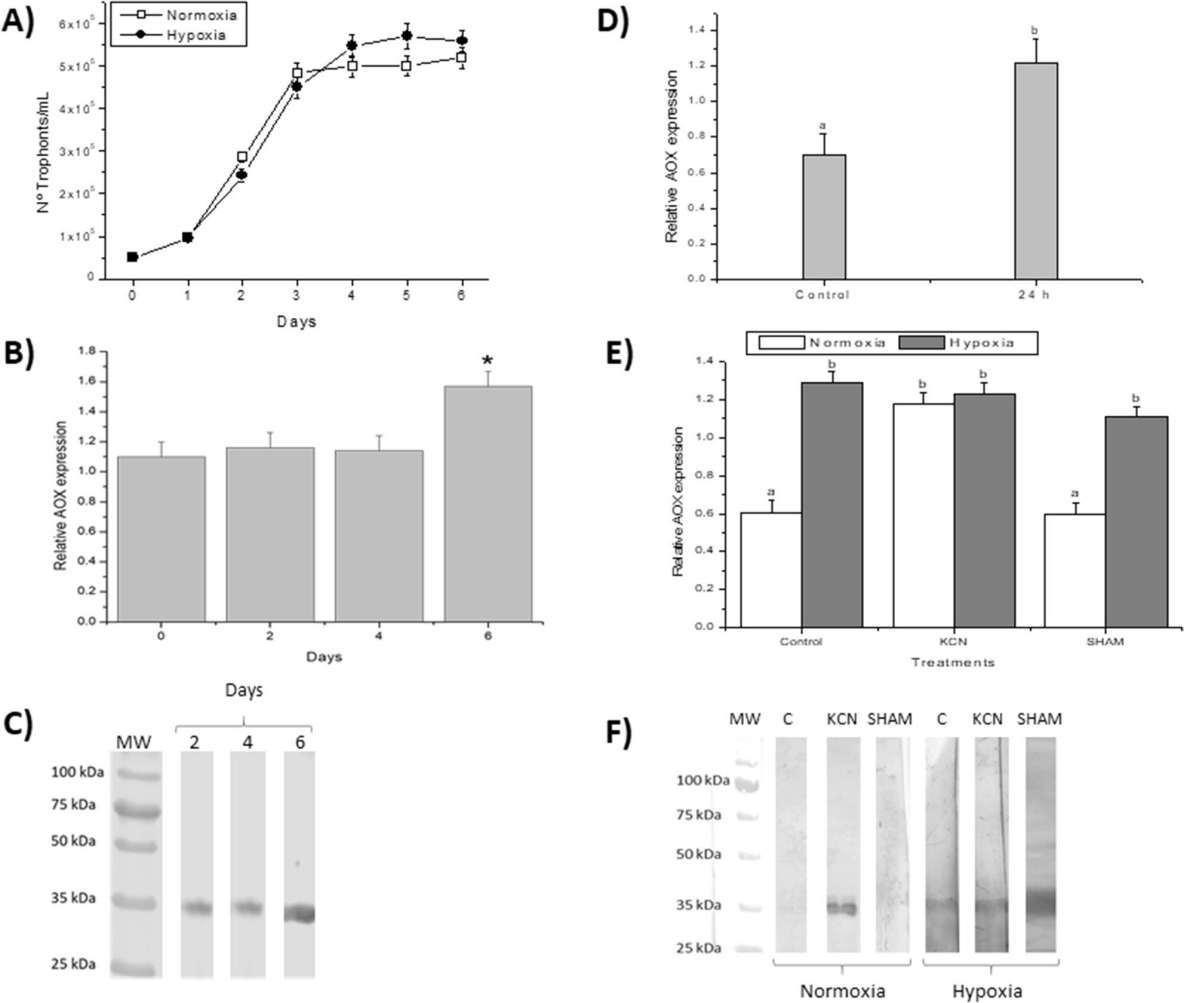
Analysis of the expression of the *P. dicentrarchi* AOX gene. (**A**) Ciliate growth kinetics under conditions of normoxia and hypoxia. (**B**) AOX gene transcription levels in trophonts maintained under normoxia for 6 days. (**C**) Western blot analysis using a polyclonal anti-rAOX antibody and ciliated lysates obtained on different days from a normoxic culture. (**D**) mRNA levels in trophonts obtained from in vitro cultures under normoxia (control) conditions, and in the same ciliates after intraperitoneal injection in turbot and subsequent extraction at 24 h post-infection (24 h). (**E**) AOX transcript amount in trophonts maintained under normoxia and hypoxia for 6 days (Control, **C**) and treated with 1 mM of KCN or salicylhydroxamic acid (SHAM). (**F**) Western blot analysis with a polyclonal anti-rAOX antibody in ciliate lysate of trophonts maintained in the same conditions as in (**E**). The results presented in the graphs correspond to the mean values ± the standard error (SE; n = 5). Statistically significant difference between groups are indicated by different letters (P < 0.05).

We first monitored the growth kinetics of *P. dicentrarchi* exposed to normoxia and hypoxia conditions. Growth of the ciliate was not altered under either condition (Fig. 5A), clearly indicating that the ciliate can grow normally under hypoxic conditions. We then analyzed AOX gene expression during different phases of growth under normoxia conditions, observing that the levels of transcripts produced under these circumstances were maintained at constant levels during the first 4 days of ciliate growth (i.e. during the logarithmic phase of growth). However, when ciliate growth reached the stationary phase, the levels AOX transcript amount increased significantly (Fig. 5B). The same was observed when the AOX protein expression was analyzed by Western blot with the polyclonal anti-rAOX antibody (Fig. 5C).

AOX gene expression during an infection in turbot was also analyzed. Turbot were experimentally infected with ciliates from normoxic cultures in the logarithmic phase of growth, and expression of the AOX gene was determined in the ciliates obtained from fish peritoneal cavity at 24 h post-infection (Fig. 5D). AOX expression was higher in ciliates obtained from infected fish than in ciliates maintained in vitro under normoxia conditions (Fig. 5D).

Finally, we carried out an experiment in which expression of the AOX gene was analyzed in ciliates maintained under normoxia and hypoxia and treated with cyanide and SHAM, classical inhibitors of mitochondrial CP and AP, respectively. The AOX transcript amount under hypoxia were significantly higher than under normoxia (Fig. 5E). Similarly, in presence of SHAM, AOX gene expression was also higher in hypoxic conditions (Fig. 5E). However, in presence of cyanide, the AOX expression levels were similar under both normoxia and hypoxia (Fig. 5E). The gene expression results were similar to those obtained by Western blot, after analysis of the AOX protein levels in the ciliates by using a polyclonal anti-rAOX antibody (Fig. 5F).

## Discussion

AOX is a terminal ubiquinol oxidase that participates in an alternative mitochondrial pathway that couples oxidation of ubiquinol to the reduction of O_2_ to H_2_O without contributing to the formation of an electrochemical gradient^18^. The first evidence for the existence of an AOX was based exclusively on physiological studies in thermogenic plant tissues, in which mitochondrial respiration resistant to inhibitors of the classical cytochrome pathway was attributed to the presence of cyanide-insensitive terminal oxidase^22,65^. The subsequent use of mitochondrial purification techniques enabled isolation and purification of the AOX protein and generation of mono- and polyclonal antibodies, which led to the identification of AOX by immunochemical techniques^4^. In recent years, molecular techniques have enabled identification of the genes that encode AOX and confirmation of the existence of this enzyme in numerous organisms. These techniques have also enabled study of the domains involved in enzymatic catalysis and of the topography of the mitochondrial membrane enzyme and its post-translational regulation, as well as reconstruction of the evolution of the enzyme in different taxonomic groups^21^. The genes encoding AOX have been found in all higher plants and in some algae, fungi, protists and invertebrates, but not in vertebrates^14,15,22,66,67^. We have found evidence of the existence of an alternative oxidase pathway in the mitochondria of the fish parasite *P. dicentrarchi*, by using functional physiological techniques, which measure oxygen consumption after the addition of inhibitors of the cytochrome pathway, and immunochemical assays, which use polyclonal antibodies against a fully conserved C-terminal consensus motif from isoforms 1 and 2 of plant AOX^13^. However, until now we did not have any information about the molecular characteristics of this enzyme in *P. dicentrarchi*. After assembly of the genome and transcriptome of *P. dicentrarchi* by next-generation sequencing (NGS), we used bioinformatic tools to identify and predict the structure of the gene and the mRNA that encodes the AOX. The AOX of *P. dicentrarchi* is encoded by a single nuclear gene consisting of 4 exons and 3 introns. In many plant species, AOX enzymes are encoded by a multigene family^68^; however, in fungi and in some protozoa the genes encoding AOX are less diverse than in plants and, in many cases, this enzyme is encoded by a single nuclear gene^69^. The typical structure of the AOX gene is characterized by the presence of four exons separated by three gene-specific introns of different lengths^70,71^. After transcription and splicing, the AOX gene in *P. dicentrarchi* generated a mature mRNA that encodes a protein of an estimated molecular weight of 35,638.68 daltons, a size like the standard sizes of the AOXs of various organisms ranging between 32–36 kDa^15^. AOX is an integral interfacial membrane protein that interacts with a single leaflet of the lipid bilayer^26^. The first studies of the membrane localization of AOX in plant mitochondria showed that AOX has two transmembrane helices and a short loop located in the matrix^72,73^. However, later topological studies indicated that AOX is an integral monotopic membrane protein that embeds into a single face (inner) of the membrane^74–76^. There is some controversy regarding prediction of the transmembrane topology of the AOX in *P. dicentrarchi*. According to the Phobius program, AOX has two transmembrane helix regions, while other bioinformatic programs predict a single transmembrane helix region. In the first case, it is assumed that the C- and N-terminal domains of the protein will be located in the mitochondrial matrix; however, in the second model the N-terminal motif would be located outside the inner membrane and the C-terminal towards the mitochondrial matrix^77^. To resolve these differences, new computational methods are probably needed, as suggested by other authors^78^. In any case, until the crystal structure of AOX of *P. dicentrarchi* is available, it will not be possible to definitively clarify the topology of this enzyme.

Analysis of AOX crystal structure indicates that it is a homodimer with a non-haem diiron carboxylate protein in which the metal atoms are ligated to four fully conserved glutamate and histidine residues^22, 79–81^. Although the AOX family contains highly conserved active sites, small differences have been observed in ligands associated with the active center in several AOXs^22^. *P. dicentrarchi* has the typical C-terminal E-X-X-H motif^73,82^, in which the conserved glutamate and histidine residues participate in the coordination of two iron atoms^21^.

Phylogenetic studies of AOX sequences indicate that Viridiplantae, Metazoan, Fungi and SAR form monophyletic clades^25,83^. On the other hand, Maximum Likelihood methods have revealed the existence of two main clades of AOX proteins: a clade containing AOXs of Ascomycota and Basidiomycota fungi, invertebrates, kinetoplastids and alveolate protozoa, and another clade that includes plants, algae and various fungal groups^83^. In our study, phylogenetic analysis by ML and BI methods also indicated the existence of two main clades of AOXs: and Sar (apicomplexans and kinetoplastids), and another including Metazoa, Fungi and Sar (Ciliophora). The present results also indicate that the AOX of *P. dicentrarchi* is closely related to AOXs of other ciliate species, especially the scuticociliate *P. persalinus*, but it is relatively distant from other Sar members as apicomplexans and kinetoplastids. In addition, although the enzyme sequence is highly conserved among strains of *P. dicentrarchi*, some differences could be used, together with sequences of other proteins, to carry out multigenic genotyping, to enable accurate, intraspecific characterization of the isolates of this scuticociliate^84^.

Plant studies indicate that AOX is an integral homodimeric membrane protein in which the monomers have an estimated molecular mass of 40 kDa and a mitochondrial directed sequence that is cleaved to generate mature forms with a molecular mass of over 32 kDa^75^. Immunochemical and immunohistochemical analysis indicates that *P. dicentrarchi* have monomeric forms (of about 35 kDa) and homodimeric forms (of 70 kDa) associated with the mitochondrial inner membrane.

These results strongly suggest that a disulfide bond can link two monomers (there are some Cys residues that could be involved, eg. Cyst77 of AOX sequence). This is what occurs in plants, where the status of this disulfide bond impacts in enzyme activity, thus when there is not disulfide bond between two monomers (reduced form), the AOX activity is strongly activated by allosteric effectors, while the oxidized form (disulfide bond between two monomers) has little activity, even with high concentrations of the allosteric effectors^79,85^. Therefore, the results obtained indicate that *P. dicentrarchi* may show two AOX forms: one monomeric of 35 kDa and other dimeric of 70 kDa, due to the presence of a mixture of “reduced” and “oxidized” forms in the mitochondrial membrane.

Phylogenetic analysis of AOX from different organisms has indicated the existence of a monophyletic clade among benthic marine animals and the presence of AOX in these species, which is probably related to a mechanism of adaptation to variations in oxygen concentration, hypoxic conditions and increased hydrogen sulphide concentration^86–88^. This hypothesis can also be extended to *P. dicentrarchi*, a benthic (like most scuticociliates) marine organism, which is also microaerophilic^13,89^.

Expression of AOX has been reported to be modulated in response to hypoxia and anoxia or after reoxygenation^18^. AOX has a lower affinity for O_2_ than cytochrome oxidase, possibly indicating higher expression/activity under conditions of low oxygen concentration^90,91^. It has also been suggested that AOX expression can be increased in plants under hypoxia to reduce the formation of superoxide (O_2_^−^), nitric oxide (NO) and peroxynitrite (ONOO^-^), as well as to reduce the toxic effect of H_2_O_2_^92–94^. *P. dicentrarchi* is a microaerophilic microorganism capable of surviving in conditions of low oxygen concentrations^13^. Under these conditions, amount of AOX transcript and of protein increase significantly in the ciliate. Gene transcription and protein expression increase during the stationary phase of growth, during which O_2_ levels in the culture are low^13^. The low concentrations of O_2_ and the increased levels of reactive oxygen species (ROS) are also associated with infection in the host. In the first case, O_2_ levels during the endoparasitic phase are low, while ROS levels are high as a result of the innate immune response produced during infection^95,96^. Both the infection and the innate humoral immune response generated by the host induce a significant increase in AOX transcription in the ciliate.

Agents that inhibit the cytochrome bc1 significantly induce AOX transcription^97,98^. Hypoxia, generated either by the removal of O_2_ from the culture medium or by the addition of cyanide, significantly induces the transcription and expression of AOX^13^. On the other hand, the addition of the AOX inhibitor, SHAM^99^, only increases the transcription of the enzyme under hypoxic conditions. The data of the increase in AOX transcription obtained in this study are closely related to the results obtained at the physiological level. Thus, the determination of the mitochondrial oxygen consumption shows that under hypoxia conditions and in the presence of inhibitors of the cytochrome pathway, the ciliate preferably uses the alternative pathway respiration^13^.

In conclusion, we have identified the gene and the mRNA that encode a protein belonging to the AOX family, not previously characterized at the molecular level in the scuticociliate *P. dicentrarchi*. In this ciliate, the AOX occurs in the monomeric and dimeric forms and is associated with the inner membrane of the mitochondria. The amino acid sequence was found to be highly conserved among the different strains of this ciliate and is closely related to AOXs of ciliate species and, particularly, to the AOX of scuticociliates. Gene transcription increased significantly under conditions of hypoxia and after infection and addition of inhibitors of cytochrome pathway respiration. The molecular characterization of AOX, together with the functional evidence of its role in adapting to parasitism and in resistance to oxidative stress induced after infection, opens a promising route for developing structure-based antiparasitic drug design strategies through molecular docking.

## Supplementary information

Supplementary information

## Data Availability

The datasets generated during and/or analysed during the current study are available from the corresponding author on reasonable request.

## References

[1] Moore AL, Albury MS (2008). Further insights into the structure of the alternative oxidase: From plants to parasites. Biochem. Soc. Trans..

[2] Albury MS, Elliott C, Moore AL (2009). Towards a structural elucidation of the alternative oxidase in plants. Physiol. Plant..

[3] Ünlü ES, Ünüvar ÖC, Aydın M (2019). Identification of alternative oxidase encoding genes in *Caulerpa cylindracea* by de novo RNA-Seq assembly analysis. Mar. Genom..

[4] Lambowitz AM, Sabourin JR, Bertrand H, Nickels R, McIntosh L (1989). Immunological identification of the alternative oxidase of *Neurospora crassa* mitochondria. Mol. Cell. Biol..

[5] Li Q (1996). Cloning and analysis of the alternative oxidase gene of *Neurospora crassa*. Genetics.

[6] Veiga A, Arrabaca JD, Loureiro-Dias MC (2003). Cyanide-resistant respiration, a very frequent metabolic pathway in yeasts. FEMS Yeast Res..

[7] Juarez O (2006). The physiologic role of alternative oxidase in *Ustilago maydis*. FEBS J..

[8] Williams BA (2010). A broad distribution of the alternative oxidase in microsporidian parasites. PLoS Pathog..

[9] Luévano-Martínez LA (2019). Mitochondrial alternative oxidase is determinant for growth and sporulation in the early diverging fungus *Blastocladiella emersoni*. Fungal Biol..

[10] Jarmuszkiewicz W, Wagner AM, Wagner MJ, Hryniewiecka L (1997). Immunological identification of the alternative oxidase of *Acanthamoeba castellanii* mitochondria. FEBS Lett..

[11] Castro-Guerrero NA, Krab K, Moreno-Sánchez R (2004). The alternative respiratory pathway of *Euglena* mitochondria. J. Bioenergy Biomembr..

[12] Chaudhuri M, Ott RD, Hill GC (2006). *Trypanosome* alternative oxidase: From molecule to function. Trends Parasitol..

[13] Mallo N, Lamas J, Leiro JM (2013). Evidence of an alternative oxidase pathway for mitochondrial respiration in the scuticociliate *Philasterides dicentrarchi*. Protist.

[14] McDonald AE, Vanlerberghe GC, Staples JF (2009). Alternative oxidase in animals: Unique characteristics and taxonomic distribution. J. Exp. Biol..

[15] Araújo Castro J, Gomes Ferreira MD, Santana Silva RJ, Andrade BS, Micheli F (2017). Alternative oxidase (AOX) constitutes a small family of proteins in *Citrus clementina* and *Citrus sinensis* L. Osb. PLoS ONE.

[16] Moore AL, Shiba T, Young L, Harada S, Kita K, Ito K (2013). Unraveling the heater: New insights into the structure of the alternative oxidase. Annu. Rev. Plant Biol..

[17] Kumari A, Pathak PK, Bulle M, Igamberdiev AU, Gupta KJ (2019). Alternative oxidase is an important player in the regulation of nitric oxide levels under normoxic and hypoxic conditions in plants. J. Exp. Bot..

[18] Vanlerberghe GC (2013). Alternative oxidase: A mitochondrial respiratory pathway to maintain metabolic and signaling homeostasis during abiotic and biotic stress in plants. Int. J. Mol. Sci..

[19] Pu X (2015). Roles of mitochondrial energy dissipation systems in plant development and acclimation to stress. Ann Bot..

[20] Walker R, Saha L, Hill GC, Chaudhuri M (2005). The effect of over-expression of the alternative oxidase in the procyclic forms of* Trypanosoma brucei*. Mol. Biochem. Parasitol..

[21] Rogov AG, Sukhanova EI, Uralskaya LA, Aliverdieva DA, Zvyagilskaya RA (2014). Alternatiive oxidase: Distribution, induction, properties, structure, regulation, and functions. Biochemistry (Moscow).

[22] May B, Young L, Moore AL (2017). Structural insights into the alternative oxidases: Are all oxidases made equal?. Biochem. Soc. Trans..

[23] Antos-Krzeminska N, Jarmuszkiewicz W (2014). Functional expression of the *Acanthamoeba castellanii* alternative oxidase in *Escherichia coli*; regulation of the activity and evidence for AcAox gene function. Biochem. Cell Biol..

[24] Henriquez FL (2009). *Acanthamoeba* alternative oxidase genes: Identification, characterization and potential as antimicrobial targets. Int. J. Parasitol..

[25] Kimura K, Kuwayama H, Amagai A, Maeda Y (2010). Developmental significance of cyanide-resistant respiration under stressed conditions: Experiments in *Dictyostelium* cells. Dev. Growth Differ..

[26] Kido Y (2010). Purification and kinetic characterization of recombinant alternative oxidase from *Trypanosoma brucei brucei*. Biochim. Biophys. Acta.

[27] Suzuki T (2005). Alternative oxidase (AOX) genes of African trypanosomes: Phylogeny and evolution of AOX and plastid terminal oxidase families. J. Eukaryot. Microbiol..

[28] Murphy AD, Lang-Unnasch N (1999). Alternative oxidase inhibitors potentiate the activity of atovaquone against *Plasmodium falciparum*. Antimicrob. Agents Chemother..

[29] Suzuki T (2004). Direct evidence for cyanide-insensitive quinol oxidase (alternative oxidase) in apicomplexan parasite *Cryptosporidium parvum*: Phylogenetic and therapeutic implications. Biochem. Biophys. Res. Commun..

[30] Sharpless TK, Butow RA (1970). An inducible alternate terminal oxidase in *Euglena gracilis* mitochondria. J. Biol. Chem..

[31] Doussiere J, Vignais PV (1984). AMP-dependence of the cyanide-insensitive pathway in the respiratory chain of *Paramecium tetraurelia*. Biochem. J..

[32] Young PG (1983). The SHAM-sensitive alternative oxidase in *Tetrahymena pyriformis*: Activity as a function of growth state and chloramphenicol treatment. J. Gen. Microbiol..

[33] Coyne RS (2011). Comparative genomics of the pathogenic ciliate *Ichthyophthirius multifiliis*, its free-living relatives and a host species provide insights into adoption of parasitic lifestyle and prospects for disease control. Genome Biol..

[34] Xiong J (2015). Genome of the facultative scuticociliatosis pathogen *Pseudocohnilembus persalinus* provides insight into its virulence through horizontal gene transfer. Sci. Rep..

[35] Mallo N, Lamas J, Leiro JM (2014). Alternative oxidase inhibitors as antiparasitic agents against scuticociliatosis. Parasitology.

[36] Morais P, Piazzon C, Lamas J, Mallo N, Leiro JM (2013). Effect of resveratrol on oxygen consumption by *Philasterides dicentrarchi*, a scuticociliate parasite of turbot. Protist.

[37] Iglesias R (2001). *Philasterides dicentrarchi* (Ciliophora, Scuticociliatida) as the causative agent of scuticociliatosis in farmed turbot *Scophthalmus maximus* in Galicia (NW Spain). Dis. Aquat. Organ..

[38] Paramá A (2003). *Philasterides dicentrarchi* (Ciliophora, Scuticociliatida): Experimental infection and possible routes of entry in farmed turbot (*Scophthalmus maximus*). Aquaculture.

[39] Iglesias R (2003). In vitro growth requeriments for the fish pathogen *Philasterides dicentrarchi* (Ciliophora, Scuticociliatida). Vet. Parasitol..

[40] Jansen HJ (2017). Rapid de novo assembly of the European eel genome from nanopore sequencing reads. Sci. Rep..

[41] Haas (2013). *De novo* transcript sequence reconstruction from RNA-seq using the Trinity platform for reference generation and analysis. Nat. Protoc..

[42] Folgueira I, Lamas J, de Felipe AP, Sueiro RA, Leiro JM (2019). Identification and molecular characterization of superoxide dismutases isolated from a scuticociliate parasite: Physiological role in oxidative stress. Sci. Rep..

[43] Barua S, Das B (2016). Preparation and characterization of chitosan-based hydrogel. World J. Pharm. Pharm. Sci..

[44] Iglesias R (2003). *Philasterides dicentrarchi* (Ciliophora: Scuticociliatida) expresses surface immobilization antigens that probably induce protective immune responses in turbot. Parasitology.

[45] Mallo N, Lamas J, Piazzon C, Leiro J (2015). Presence of a plant-like proton-translocating pyrophosphatase in a scuticociliate parasite and its role as a possible drug target. Parasitology.

[46] Mallo N (2016). Role of H^+^-pyrophosphatase activity in the regulation of intracellular pH in a scuticociliate parasite of turbot: Physiological effects. Exp. Parasitol..

[47] Paramá A, Arranz JA, Alvarez MF, Sanmartín ML, Leiro J (2006). Ultrastructure and phylogeny of *Philasterides dicentrarchi* (Ciliophora, Scuticociliatia) from farmed turbot in NW Spain. Parasitology.

[48] Iglesias R, Paramá A, Álvarez MF, Leiro J, Sanmartín ML (2002). Antiprotozoals effective in vitro against the scuticociliate fish pathogen *Philasterides dicentrarchi*. Dis. Aquat. Org..

[49] Bustin SA (2009). The MIQE guidelines: minimum information for publication of quantitative real-time PCR experiments. Clin Chem..

[50] Mitchell AL (2019). Improving coverage, classification and access to protein sequence annotations. Nucleic Acids Res..

[51] Käll L, Krogh A, Sonnhammer ELL (2004). A combined transmembrane topology and signal peptide prediction method. J. Mol. Biol..

[52] Claros MG, Vincens P (1996). Computational method to predict mitochondrially imported proteins and their targeting sequences. Eur. J. Biochem..

[53] Gasteiger E, Walker JM (2005). Protein identification and analysis tools on the ExPASy server. The Proteomics Protocols Handbook.

[54] Gupta R, Brunak S (2002). Prediction of glycosylation across the human proteome and the correlation to protein function, pacific symposium on biocomputing. Pac. Symp. Biocomput..

[55] Wang J, Torii M, Liu H, Hart GW, Hu ZZ (2011). dbOGAP: An integrated bioinformatics resource for protein O-GlcNAcylation. BMC Bioinform..

[56] Omasits U, Ahrens CH, Müller S, Wollscheid B (2014). Protter: Interactive protein feature visualization and integration with experimental proteomic data. Bioinformatics.

[57] Goldberg T (2014). LocTree3 prediction of localization. Nucleic Acids Res..

[58] Waterhouse A (2018). SWISS-MODEL: Homology modelling of protein structures and complexes. Nucleic Acids Res..

[59] Madeira F (2019). The EMBL-EBI search and sequence analysis tools APIs in 2019. Nucleic Acids Res..

[60] Capella-Gutiérrez S, Silla-Martínez JM, Galbaldón T (2009). trimAl: A tool for automated alignment trimming in large-scale phylogenetic analyses. Bioinformatics.

[61] Jones DT, Taylor WR, Thornton JM (1992). The rapid generation of mutation data matrices from protein sequences. Comput. Appl. Biosci..

[62] Hillis DM, Bull JJ (1993). An empirical test of bootstrapping as a method for assessing confidence in phylogenetic analysis. Syst. Biol..

[63] Kumar S, Stecher G, Li M, Knyaz C, Tamura K (2018). MEGA X: Molecular evolutionary genetics analysis across computing platforms. Mol. Biol. Evol..

[64] Huelsenbeck JP, Ronquist F (2001). MRBAYES: Bayesian inference of phylogenetic trees. Bioinformatics.

[65] Meeuse BJ (1975). Thermogenic respiration in aroids. Annu. Rev. Plant Physiol. Plant Mol. Biol..

[66] McDonald AE, Vanlerberghe GC (2006). Origins, evolutionary history, and taxonomic distribution of alternative oxidase and plastoquinol terminal oxidase. Physiol. Part D Genom. Proteomics.

[67] Rodríguez-Armenta C (2018). Alternative mitochondrial respiratory chains from two crustaceans: *Artemia franciscana* nauplii and the white shrimp, *Litopenaeus vannamei*. J. Bioenergy Biomembr..

[68] Dinant M, Baurain D, Coosemans N, Joris B, Matagne RF (2001). Characterization of two genes encoding the mitochondrial alternative oxidase in *Chlamydomonas reinhardtii*. Curr. Genet..

[69] Roberts CW (2004). Evidence for mitochondrial-derived alternative oxidase in the apicomplexan parasite *Cryptosporidium parvum*: A potential anti-microbial agent target. Int. J. Parasitol..

[70] Polidoros AN, Mylona PV, Arnholdt-Schmitt B (2009). Aox gene structure, transcript variation and expression in plants. Physiol. Plant..

[71] Costa JH (2010). Stress-induced co-expression of two alternative oxidase (VuAox1 and 2b) genes in *Vigna unguiculata*. J. Plant Physiol..

[72] Umbach AL, Siedow JN (2000). The cyanide-resistant alternative oxidases from the fungi *Pichia stipitis* and *Neurospora crassa* are monomeric and lack regulatory features of the plant enzyme. Arch. Biochem. Biophys..

[73] Siedow JN, Umbach AL, Moore AL (1995). The active site of the cyanide-resistant oxidase from plant mitochondria contains a binuclear iron center. FEBS Lett..

[74] Anderson ME, Nordlund P (1999). A revised model of the active site of alternative oxidase. FEBS Lett..

[75] Berthold DA, Andersson ME, Nordlund P (2000). New insight into the structure and function of the alternative oxidase. Biochim. Biophys. Acta.

[76] Young L (2013). The alternative oxidases: Simple oxidoreductase proteins with complex functions. Biochem. Soc. Trans..

[77] Bonnefoy N, Fiumera HL, Dujardin G, Fox TD (2009). Roles of Oxa1-related inner-membrane translocases in assembly of respiratory chain complexes. Biochem. Biophys. Acta.

[78] Allen KN, Entova S, Ray LC, Imperiali B (2019). Monotopic membrane proteins join the fold. Trends Biochem. Sci..

[79] Umbach AL, Siedow JN (1993). Covalent and noncovalent dimers of the cyanide-resistant alternative oxidase protein in higher plant mitochondria and their relationship to enzyme activity. Plant Physiol..

[80] Berthold DA, Stenmark P (2003). Membrane-bound di-iron carboxylate proteins. Ann. Rev. Plant Biol..

[81] Shiba T (2013). Structure of the *Trypanosome* cyanide-insensitive alternative oxidase. Proc. Natl. Acad. Sci. U.S.A..

[82] Moore AL, Umbach AL, Siedow JN (1995). Structure-function relationships of the alternative oxidase of plant mitochondria: A model of the active site. J. Bioenergy Biomembr..

[83] Pennisi R (2016). Molecular evolution of alternative oxidase proteins: A phylogenetic and structure modelling approach. J. Mol. Evol..

[84] De Felipe AP, Lamas J, Sueiro RA, Folgueira I, Leiro JM (2017). New data on flatfish scuticociliatosis reveal that *Miamiensis avidus* and *Philasterides dicentrarchi* are different species. Parasitology.

[85] Umbach AL, Ng VS, Siedow JN (2006). Regulation of plant alternative oxidase activity: A tale of two cysteines. Biochim. Biophys Acta.

[86] Turrens JF (2003). Mitochondrial formation of reactive oxygen species. J. Physiol..

[87] Abele E, Philip E, González PM, Puntarulo S (2007). Marine invertebrate mitochondria and oxidative stress. Front. Biosci..

[88] Sussarellu R (2013). Rapid mitochondrial adjustments in response to short term hypoxia and re-oxygenation in the Pacific oyster, *Crassostrea gigas*. J. Exp. Biol..

[89] Hayward BH, Droste R, Epstein SS (2003). Interstitial ciliates: benthic microaerophiles or plarktonic anaerobes?. J. Eukaryot. Microbiol..

[90] Millar AH, Bergersen FJ, Day DA (1994). Oxygen affinity of terminal oxidases in soybean mitochondria. Plant Physiol. Biochem..

[91] Ribas-Carbo M, Berry JA, Azcon-Bieto J, Siedow JN (1994). The reaction of the plant mitochondrial cyanide-resistant alternative oxidase with oxygen. Biochim. Biophys. Acta.

[92] Amor Y, Chevion M, Levine A (2000). Anoxia pretreatment protects soybean cells against H_2_O_2_-induced cell death: possible involvement of peroxidases and of alternative oxidase. FEBS Lett..

[93] Igamberdiev AU, Ratcliffe RG, Gupta KJ (2014). Plant mitochondria: Source and target for nitric oxide. Mitochondrion.

[94] Vishwakarma A, Kumari A, Mur LAJ, Gupta KJ (2018). A discrete role for alternative oxidase under hypoxia to increase nitric oxide and drive energy production. Free Radic. Biol. Med..

[95] Leiro J, Arranz JA, Iglesias R, Ubeira FM, Sanmartín ML (2004). Effects of the histiophagous ciliate *Philasterides dicentrarchi* on turbot phagocyte responses. Fish Shellfish Immunol..

[96] Piazzon MC, Wiegertjes GF, Leiro J, Lamas J (2011). Turbot resistance to *Philasterides dicentrarchi* is more dependent on humoral than on cellular immune responses. Fish Shellfish Immunol..

[97] Yukioka H (1998). Transcriptional activation of the alternative oxidase gene of the fungus *Magnaporthe grisea* by a respiratory-inhibiting fungicide and hydrogen peroxide. Biochim. Biophys. Acta.

[98] Fu LJ (2010). Systemic induction and role of mitochondrial alternative oxidase and nitric oxide in a compatible tomato-Tobacco mosaic virus interaction. Mol. Plant Microbe Interact..

[99] Ebiloma GU, Balogun EO, Cueto-Díaz EJ, de Koning HP, Dardonville C (2019). Alternative oxidase inhibitors: Mitochondrion-targeting as a strategy for new drugs against pathogenic parasites and fungi. Med. Res. Rev..

