# Supplementary material for: Molecular characterization and gene expression modulation of the alternative oxidase in a scuticociliate parasite by hypoxia and mitochondrial respiration inhibitors
Source: Sci Rep. 2020 Jul 17;10:11880. doi: 10.1038/s41598-020-68791-9 (PMC7367826; doi:10.1038/s41598-020-68791-9)
Supplement: Supplementary file 1 — Supplementary information [file 41598_2020_68791_MOESM1_ESM.docx]

Molecular characterization and gene expression modulation of the alternative oxidase in a scuticociliate parasite by hypoxia and mitochondrial respiration inhibitors

Iría Folgueira., Lamas, J., Sueiro, R.A., Leiro, J.


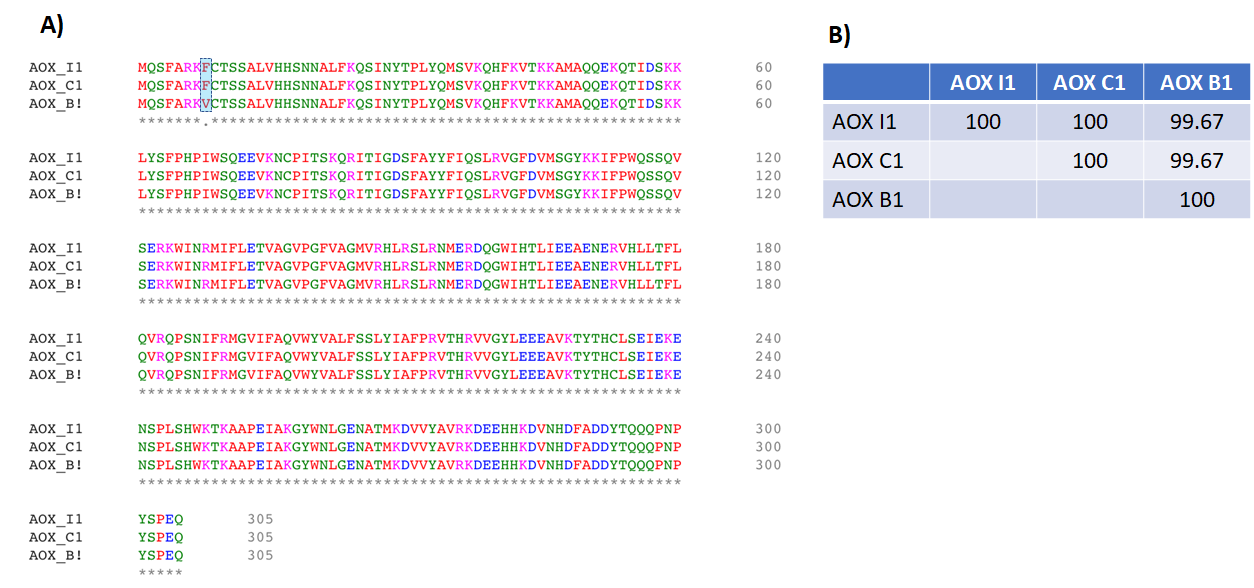


**Supplementary information. -** A) Alignment of the amino acid sequences corresponding to three *P. dicentrarchi* isolates (I1, B1 and C1)*.* Conserved amino acid sites are indicated by a star and semi-conserved sites, by dots. B) Percent identity matrix created with ClustalW 2.1.


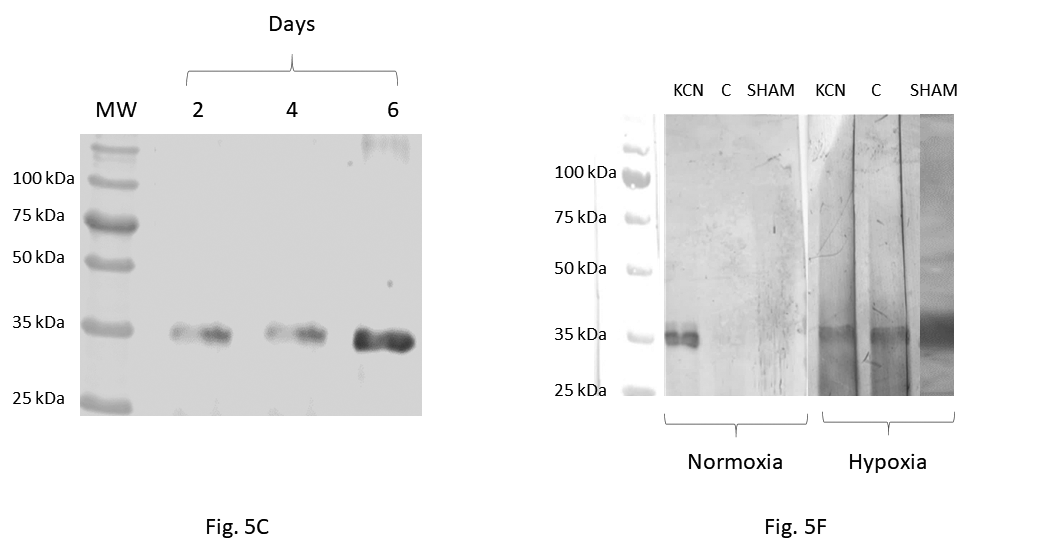


**Supplementary information. -** Complete original blots corresponding to Fig. 5C and 5F of the manuscript.
